# Micellar Solubilization of Phenols With One or Two Hydroxyl Groups Using Biological Surfactant Rhamnolipid

**DOI:** 10.1002/mrc.5530

**Published:** 2025-05-19

**Authors:** Victor P. Arkhipov, Ruslan V. Arkhipov, Andrei Filippov

**Affiliations:** ^1^ Department of Physics Kazan National Research Technological University Kazan Russian Federation; ^2^ Institute of Physics, Kazan Federal University Kazan Russian Federation; ^3^ Chemistry of Interfaces Luleå University of Technology Luleå Sweden

**Keywords:** diffusometry, micelles, NMR, phenols, rhamnolipid, solubilization

## Abstract

We studied the solubilization of phenols with one and two hydroxyl groups (phenol, p‐cresol, guaiacol, and pyrocatechol, resorcinol, hydroquinone) by micelles of the biological surfactant rhamnolipid using NMR diffusometry. We discuss the results within the framework of a model of two states of solubilizer molecules in solution: free in the aqueous phase and bound in surfactant micelles. The solubilization characteristics of rhamnolipid were calculated: the proportion of solubilized molecules р, micelle‐water partition coefficient *K*_*m*_, and molar solubilization ratio (MSR) depending on the concentration of rhamnolipid in solutions.

## Graphical abstract

Solubilization of phenols with one and two hydroxyl groups by micelles of the biological surfactant rhamnolipid was studied by NMR diffusometry.

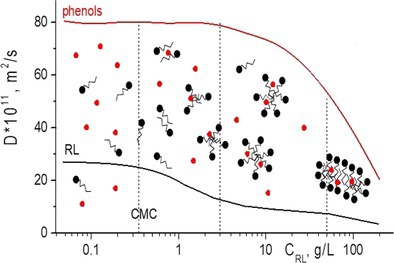

## 1 Introduction

The pressing environmental problem of our time is the protection of the environment: soil, water, and air from pollution [1]. Water and soil are polluted by heavy metals [2]; organic and inorganic compounds; and household, livestock, and municipal waste [3], which pose a threat to health and food security. Phenols and phenolic compounds, which are highly toxic substances, are widely used in industry [4] as antioxidants, chemical intermediates, disinfectants, tannins, additives to lubricants and gasoline, and in the production of artificial resins. Phenols are protoplasmic poisons and are toxic to all cells and microorganisms, which complicates the biological treatment of industrial wastewater to remove phenolic compounds. The relevance of the work is related to the problems of environmental pollution and the search for ways to clean it, methods of soil remediation, removal of organic compounds, and heavy metals from industrial wastewater.

Among the regenerative and destructive methods of soil remediation from oil and petroleum products during spills [5, 6], cleaning industrial wastewater from phenolic compounds [7, 8], an important place is occupied by methods of extraction using surfactants [9]. Extraction methods based on surfactants can be divided into methods based on cloud point (CP) extraction [10] and those based on the micellar solubilization phenomenon [11, 12, 13]. The micellar extraction method is based on the ability of surfactant micelles to dissolve organic compounds, oil fractions, metals in the form of chelate complexes, and other pollutants [14, 15, 16, 17, 18, 19, 20, 21]. The procedure for the CP preconcentration of phenol derivatives and its analogues using simple and combined systems based on nonionic and cationic surfactants is described [22]. Micellar‐enhanced ultrafiltration (MEUF) was applied to separate the phenolic compounds p‐nitrophenol, p‐chlorophenol, p‐cresol, and phenol from wastewater using cationic, anionic, and nonionic surfactants [23]. Solubilization of phenol, p‐methylphenol, p‐ethylphenol, and p‐propylphenol by mixed micelles of CTAB and copolymers of maleic acid and styrene is investigated, and the distribution coefficients of phenols between the micellar and aqueous phases are calculated [24]. The solubilization of phenol in nonionic micelles of polyethoxylated nonylphenols was studied, and the degree of partitioning of phenol into the micelles was found [14].

However, the use of synthetic surfactants itself has a negative impact on the environment, partly due to their toxicity and low biodegradability. A promising “green” alternative to synthetic surfactants is biological surfactants produced by living microorganisms [25, 26, 27]. Due to their unique physical and chemical properties, as well as antibacterial and antiviral activity, biosurfactants are used in the oil industry, agriculture, medicine, cosmetology, food industry, production of detergents and cleaning agents, textile industry, etc. [28, 29, 30].

**FIGURE 1:**
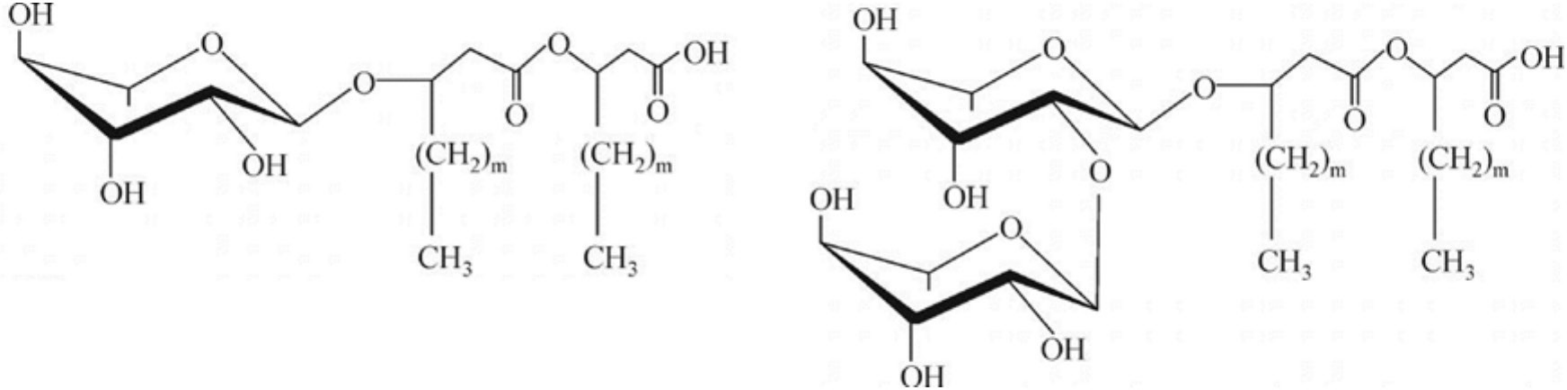
Structures of molecules of (a) mono‐rhamnolipid and (b) di‐rhamnolipid.

Rhamnose lipids or rhamnolipids [31] are biological anionic surfactants belonging to a class of glycolipids produced by *Pseudomonas aeruginosa* bacteria [32]. Rhamnolipids contain [33] a hydrophilic head of one (Rha) or two (Rha‐Rha) rhamnose groups and a hydrophobic tail of one or two 3‐hydroxy fatty acid chains (mainly C_8_, С_10_, C_12_), Figure 1. Rhamnolipids produced by bacteria are mono‐ and di‐rhamnolipids, as well as fatty acids chains, with their relative concentrations depending on the synthesis conditions [34, 35]. At concentrations above the critical micelle concentration (CMC) (0.01 ÷ 0.2 g/L) [36, 37, 38, 39], rhamnolipids form micelles and vesicles capable of solubilizing lipophilic aliphatic, aromatic, and polycyclic hydrocarbons [40, 41, 42, 43]. Rhamnolipids are weak acids, and the wide range of CMC values (1–400 mg/L) [40] recorded for mono‐ and diRL, as well as their mixtures, is explained by numerous factors that can influence the CMC: sample purity level, pH, ionic strength, and the presence of unsaturated bonds [36]. Di‐ramnolipid has a lower CMC (110 μM) than mono‐rhamnolipid (166 μM) [44]. The CMC of a rhamnolipid mixture is determined by the ratio of mono‐ and di‐rhamnolipid [35]. It was found [45] that the morphology of rhamnolipids changed from lamellar to vesicular and micellar with an increase in pH from 5.8 to 8. It has been shown [46] that rhamnolipids retain high emulsifying activity and emulsion stability over a wide pH range. The solubilization characteristics of monobasic phenol with one hydroxyl group and resorcinol with two hydroxyl groups change insignificantly [47] with pH conditions from 4 to 10.

Despite the wide range of applications of biosurfactants, the processes of micellar solubilization with their participation are poorly studied. Previously, the authors studied the solubilization properties of rhamnolipid micelles in saturated solutions of benzene, toluene, ethylbenzene, xylene [48], naphthalene [49], and phenol [50] at various concentrations. This work is a continuation of the studies initiated with the aim of studying and comparing the solubilizing properties of rhamnolipid micelles in relation to water‐soluble phenols with one hydroxyl group (phenol, p‐cresol, and guaiacol), as well as with two hydroxyl groups (pyrocatechol, resorcinol, and hydroquinone). The studies of the solubilizing properties of rhamnolipid micelles were carried out using the NMR diffusometry method [51, 52]. All measurements were performed at 25 °C.

## 2 Materials and Methods

### 2.1 Materials

RLs were purchased from Merck (Germany) produced by AGAE Technologies LLC, Corvallis, Oregon, 97333, United States. It is a mixture of mono‐ and di‐RL; the content of RLs in the powder was more than 90%. The ^13^C NMR spectra shows that the studied unfractionated rhamnolipid contains 44% mono‐ and 56% di‐RL [50]. No additional purification or fractionation were performed. All phenols were of a chemically pure grade.

The solutions for NMR measurements were prepared in deuterated water (Sigma, degree of substitution 99.9%). The pH (pD) measured on the Mettler Toledo SevenCompact pH meter gave a value of 5.67. Therefore, taking into account the corrections recommended in the works [53], pH (pD) = 5.67 + 0.44 = 6.11. The use of deuterated water made it possible to exclude the intense line of water from the 1H NMR spectra.

The concentration of phenols in all solutions was 5 g/L. The concentrations of the RL varied from C = 200 g/L to C = 0.05 g/L. Initial solutions with maximal concentrations of the RL was carefully mixed and left to equilibrate for at least 2 days. Other solutions were prepared by serial dilution.

### 2.2 NMR Diffusometry

The translational mobility of all solution components—phenols, water, and rhamnolipid—was studied by NMR using a pulsed magnetic field gradient on a Bruker AVANCE 400 III pulsed NMR spectrometer with Fourier transformation. The device was equipped with a pulsed magnetic field gradient unit with a maximum gradient value of 28 T/m. Measurements and recording of spectra were carried out on ^1^H nuclei at a resonance frequency of 400 MHz. A DIFF50 diffusion sensor and standard 5 mm sample ampoules were used. The temperature range of the diffusion sensor is from −40 to +80 °C. The sample temperature was set and controlled by the sample thermostatting unit included in the setup. The accuracy of setting and maintaining the sample temperature was ± 0.1 °C.

A stimulated echo pulse sequence was used to measure diffusion decays [54]:

(1) $$A\left(\tau ,\tau_{1},g,\delta \right)\propto \exp\left(-\frac{2\tau }{T_{2}}-\frac{\tau_{1}}{T_{1}}\right)\exp\left(-\gamma^{2}g^{2}\delta^{2}{\mathrm{Dt}}_{d}\right),$$

where *T*_1_ and *T*_2_ are spin–lattice and spin–spin relaxation times; *τ*, *τ*_1_ are time intervals in a stimulated echo sequence, *γ* is the gyromagnetic ratio for protons, *g* and *δ* are amplitude and duration of the pulsed magnetic field gradient, *D* is the self‐diffusion coefficient, *t*_*d*_ = (*Δ*—*δ/3*) is the diffusion time, *Δ* = (*τ + τ*_*1*_). The representation of the stimulated echo signal in the form of NMR frequency spectra on ^1^H nuclei was carried out for each value of the pulse gradient amplitude by means of the Fourier transform operation. Dependence of the spin echo amplitude ln (A) upon factor *γ* ^*2*^ *δ* ^*2*^ *g* ^*2*^ *t*_*d*_ (diffusion decay) was used to measure the diffusion coefficients.

We used a sine‐shape of magnetic field gradient pulses, following the recommendations of the spectrometer’s DOSY program manual (TopSpin 3.1). The recalculation [55] of the gradient parameters of the basic formula (1), obtained for the rectangular shape of gradient pulses, to another shape—sinusoidal or trapezoidal—is performed automatically using the spectrometer software.

The magnitude of the pulse gradient varied from 0 to *g*_*max*_, which was selected individually depending on the *D* values with the condition of obtaining at least 10‐fold attenuation of the echo signal. When measuring *D* of water, *g*_*max*_ = 1.3 T/m was sufficient; when measuring *D* of solubilizates and RL, *g*_*max*_ up to 7 T/m was used. The diffusion time *t*_*d*_ = 50 ms and the duration of the field gradient pulses *δ* = 1 ms were kept the same in all measurements. The number of scans (NS) was set according to the spin echo signal amplitude of solubilizers and rhamnolipid from 4 to 32, a dummy scan (DS) = 2.

The pre‐measured spin–lattice relaxation time of the oxyethylene protons of the rhamnolipid was ≈ 0.5 s; the time between successive scans was set accordingly, RT = 5 s. The processing of diffusion decays and determination of diffusion coefficients were carried out using the software Bruker TopSpin 3.5 [56]. In the spectrometer software, the procedure for processing diffusion decays does not contain an option for estimating errors in determining the self‐diffusion coefficients. For this purpose, diffusion decays were converted into the Origin program, which has such a capability. As a result, the error in estimating D did not exceed 3–5%.

## 3 Results and Discussion

### 3.1 NMR Spectrum

**FIGURE 2:**
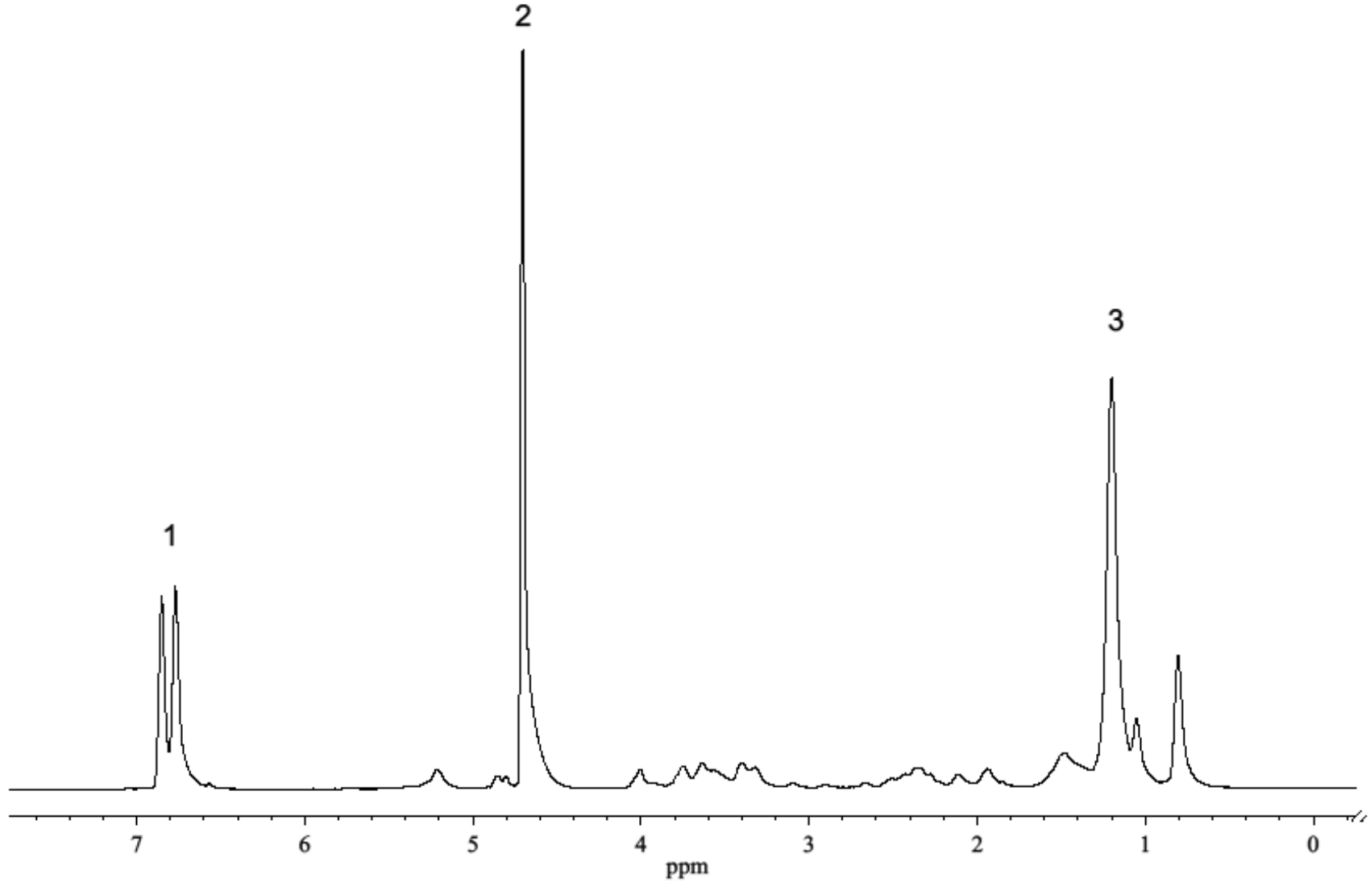
^1^H NMR spectrum of pyrocatechol (C = 5 g/L) and rhamnolipid (C = 12.5 g/L) in D_2_O: 1, line of aromatic protons of pyrocatechol; 2, line of residual water protons; 3, line of methylene protons of the RL.

The ^1^H NMR spectrum of rhamnolipid and pyrocatechol in heavy water is shown in Figure 2. The lines of aromatic (doublet δ = 6.8 ppm) protons of pyrocatechol do not overlap with the lines of rhamnolipids and residual water protons (δ = 4.7 ppm), which significantly simplifies the processing of diffusion decays. Spectra of other studied systems have a similar appearance, with no overlapping of lines. Diffusion coefficients of rhamnolipid molecules were determined from diffusion decays of the lines of fatty acid methylene protons (δ = 1.2 ppm), and phenols from decays of the lines of aromatic protons.

### 3.2 The Form of Diffusion Decay and Diffusion Coefficient

NMR diffusometry [51, 57] provides the possibility to perform selective measurements of the diffusion coefficients of molecules of all components of the solution—solvent molecules, surfactant micelles, and solubilized molecules—and then to study the dynamics of solubilization processes [58]. Comparison of the diffusion coefficients of solubilized molecules in the absence and presence of solubilization allows us to calculate the proportion of solubilized molecules and its dependence on the experimental conditions: the concentration of surfactants and solubilizer, temperature, pH of the solution, the presence of cosurfactants, and salts. Values of diffusion coefficients of phenols change by almost an order of magnitude when moving from the free state of solubilizer molecules in solution (at C_RL_<CMC) to the micelle‐bound state (at C_RL_>CMC) as a result of solubilization. NMR spectroscopy and diffusometry are informative, nondestructive methods for studying multicomponent systems; they are also used to determine the CMC of surfactant solutions based on characteristic breaks in the dependences of the magnitude of chemical shifts, the width of spectral lines, and the diffusion coefficients on the concentration of surfactant in solution [48, 50, 59]. The CMC value of the studied unfractionated rhamnolipid, not purified from impurities, determined by us in previous work [48, 49, 50] using NMR diffusometry, conductometry, and surface tension methods, is in the range of 0.17–0.35 g/L. The CMC value, determined by the inflection point of the dependence of the rhamnolipid diffusion coefficient on its concentration in solutions of all phenols, Figure 5, is 0.4 g/L.

#### 3.2.1 Lines of Water and Phenols

**FIGURE 3:**
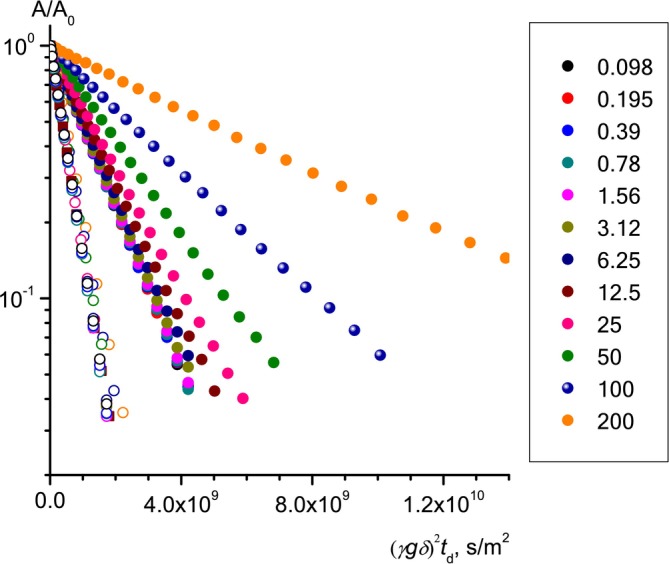
Diffusion decays of guaiacol at different RL concentrations (g/L) in D_2_O solutions.

The diffusion decays of aromatic proton lines of all the phenols studied, as well as the residual water proton lines, have a mono‐exponential character at all RL concentrations in solutions. Figure 3 shows the diffusion decays of water and guaiacol; similar diffusion decays in solutions of other phenols are presented in the Supporting Materials. Water molecules can penetrate into micelles, be in the zone of the double electric layer at the surface of micelles [60], or be in a free state, while possessing different translational mobility. Solubilizer molecules are distributed between free molecular states and those bound in micelles, which also differ in mobility. However, due to the rapid exchanges of water and solubilizer molecules between all their possible states in solution on NMR time scales, the diffusion decays of the residual water protons and aromatic solubilizer protons are single exponential, despite the different translational mobilities of their molecules in these different states.

During the measurement, the solubilizer molecules have time to be in both a free and bound state many times, and, therefore, the measured value of the self‐diffusion coefficient of the solubilizer molecules represents a weighted average value for these states [51]:

(2) $$D_{S}=\;p\cdot D_{S}^{\mathrm{mic}}+\left(1‐p\right)\cdot D_{S}^{\text{free}},$$

where p is the fraction of solubilizate molecules that are in a bound state in micelles, respectively (1‐p) is the fraction of solubilizate molecules that are in a free state in solution; $D_{S}^{\mathrm{mic}}$ and $D_{S}^{\text{free}}$ are the diffusion coefficients of solubilizate molecules that are in micelles and in a free state in solution.

Since the solubilizate molecules contained in the micelles and the micelles themselves together with the solubilizate molecules included in them, form common kinetic units, the self‐diffusion coefficient of the solubilizate molecules contained in the micelles $D_{S}^{\mathrm{mic}}$ can be taken to be equal to the measured self‐diffusion coefficient of the surfactant micelles $D_{\mathrm{RL}}^{\mathrm{mic}}$. On the other hand, it is possible to set $D_{S}^{\text{free}}$ equal to the diffusion coefficient of the solubilizate in solution, measured in the absence of surfactant or at С_surf_<CMC.

Within the framework of such obvious assumptions, it is easy to calculate the value of p from the experimentally measured self‐diffusion coefficient of the solubilizer molecules and surfactant micelles.

#### 3.2.2 Methylene Proton Line

**FIGURE 4:**
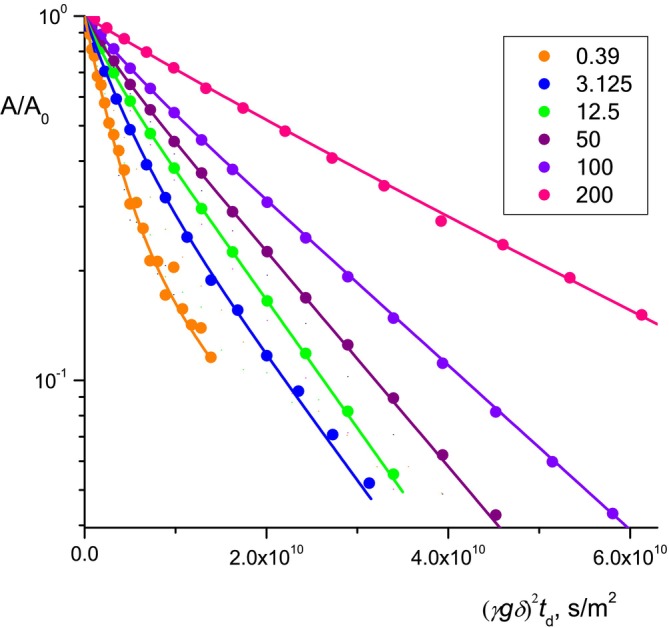
Diffusion decays of the signal of methylene protons of rhamnolipid in D_2_O solutions of hydroquinone at different RL concentrations (g/L). The lines correspond to the bi‐exponential approximation.

The methylene proton line, Figure 2, contains contributions from methylene protons of both the RL and lipid molecule residues. The form of the diffusion decays of the methylene proton lines differs from mono‐exponential and changes depending on the concentration of the RL in the solution, Figure 4. The non‐mono‐exponentiality (nonlinearity in semi‐logarithmic coordinates) of the diffusion decays of methylene protons occurs for two obvious reasons: the complex fractional composition of the RL and the presence of fatty acid residues in the solution.

At C_RL_<CMC, the nonlinearity of the diffusion decay of methylene protons is mainly due to the complex fractional composition of rhamnolipids, which is a mixture of mono‐ and di‐ rhamnolipids with variations in the length and degree of branching of fatty acid chains, mainly including Rha‐C_10_‐C_10_, Rha‐Rha‐C_10_‐C_10_ and Rha‐C_10_ [43, 48, 49], as well as the presence of associated products in the form of rhamnose residues and lipids. Previously, the authors determined by comparing the intensities of the lines of methyl carbons of lipids and rhamnose groups in the ^13^C NMR spectra [50] that the studied unfractionated rhamnolipid contains 44% mono‐ and 56% di‐RL. Due to the different translational mobility and, accordingly, different values of diffusion coefficients of different RL fractions and lipid residues, the resulting diffusion decay of the methylene proton echo signal becomes nonlinear.

At C_RL_≥CMC, mono‐ and di‐RL form mixed micelles of constant, on average, composition, determined by the ratio of mono‐ and di‐RL in the initial unfractionated RL sample. Consequently, mono‐ and di‐RL molecules located in micelles will have the same diffusion coefficient values, equal to the diffusion coefficients of the micelles themselves. Individual RL molecules present in the solution with a concentration of C = CMC also contribute to the resulting diffusion decay of the RL methylene proton line. This contribution rapidly decreases with increasing of the RL concentration and at C > 5 × CMC does not exceed 5% [61]. Considering the rapid exchange of RL molecules between the molecular and micellar states on NMR time scales, the diffusion decay of the RL methylene proton line should be single exponential. The reason for the nonlinearity observed in the initial sections, Figure 4, of the diffusion drops of methylene protons at C_RL_≥CMC is the presence of fatty acid fragments in the solution, as well as individual rhamnose groups. Therefore, at C_RL_≥CMC, the diffusion decay of the methylene protons cannot be strictly single exponential. It can be represented as the sum of two exponential components: the “long” component from the relatively slow RL micelles and the “short” component corresponding to the more mobile residues of fatty acid molecules.

Decomposition of the diffusion decay of RL methylene protons into two exponentials *A*/*A*_0_ = *p*_1_exp(‐*D*_1_ *x*) + *p*_2_exp(‐*D*_2_ *x*), where *x* = (*γgδ*)^2^ *t*_*d*_, performed using the spectrometer software, is shown in Figure 4. In particular, the self‐diffusion and population coefficients at C_RL_ = 200 g/L are equal to *D*_1_ = 7.6·10^−11^ m^2^/s (12%) and *D*_2_ = 2.9·10^−11^ m^2^/s (88%); at C_RL_ = 50 g/L are equal to *D*_1_ = 0.32·10^−11^ m^2^/s (12%) and *D*_2_ = 6.8·10^−11^ m^2^/s (88%); at C_RL_ = 3.12 g/L are equal to *D*_1_ = 0.23·10^−11^ m^2^/s (45%) and *D*_2_ = 7.8·10^−11^ m^2^/s (55%). The values of the self‐diffusion coefficients of micelles of RL determined using this approach were then used to calculate the proportion of solubilizer molecules found in micelles using the formula (3).

**FIGURE 5:**
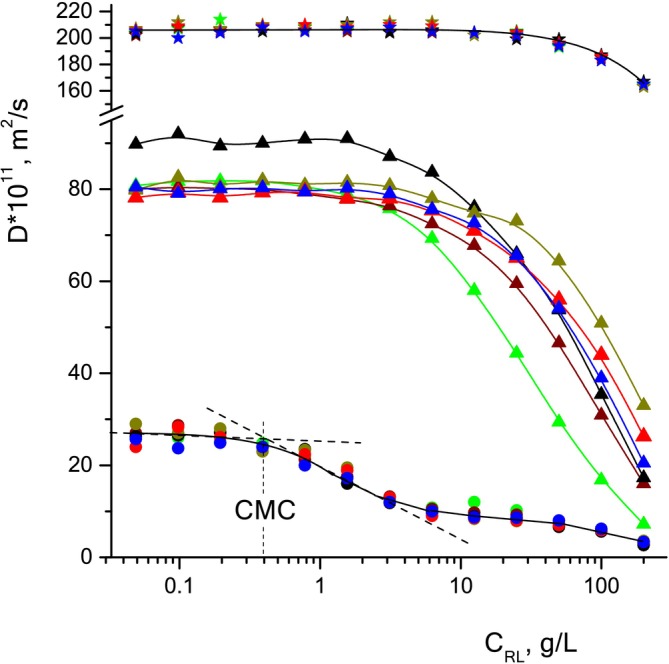
Diffusion coefficients of water molecules (star), rhamnolipid (circles), and solubilizers: phenol (black), p‐cresol (green), guaiacol (wine), pyrocatechol (blue), resorcinol (red), hydroquinone (dark yellow), respectively, depending on the RL concentration.

Figure 5 shows the results of measurements of the diffusion coefficients of molecules of individual components, solubilizers, rhamnolipid, and water, depending on the concentration of RL in solutions. As noted above, the diffusion decays of the spin echo signals of residual protons of water molecules and aromatic protons of molecules of all solubilizers were mono‐exponential at all concentrations of rhamnolipid, while the diffusion decays of RL methylene protons had a non‐mono‐exponential form. Diffusion coefficients of the RL, presented in Figure 5, were calculated: a) at C ≤ CMC for the initial sections of declines under the assumption [62] that they contain information about all possible fractions of the RL molecules; b) at C ≥ CMC without taking into account the initial sections of diffusion declines, to exclude the contribution of the “short” component, that is, the contribution from the residues of fatty acid molecules.

The values of the diffusion coefficients of water molecules in all solutions coincide with each other within the limits of possible measurement errors, and maintain a constant value of 2.06·10^−9^ m^2^/s up to concentrations of C_RL_ ≈ 20 g/L. On the curve of the dependence of the diffusion coefficient of RL on concentration, three characteristic regions can be distinguished: a) a low concentration region (С_RL_ < 0.3 g/L), which corresponds to the monomeric form of RL; b) a transition region of concentrations (0.3 g/L < С_RL_ < 2 g/L), corresponding to the formation of micelles; and c) a region (С_RL_ > 2 g/L) corresponding to the fully aggregated state of RL in solution. Dependences of the diffusion coefficients of the molecules of all the studied solubilizers have two sections: the first corresponds to the monomeric, free state of the solubilizer molecules in the solution (С_RL_ < 2 g/L); the second at С_RL_ > 2 g/L corresponds to the bound state, in which solubilizer molecules are part of the surfactant micelles. With increasing C_RL_, the values of diffusion coefficients of all solubilizates approach those of RL, which means that the efficiency of micellar solubilization increases.

### 3.3 Solubilization Parameters of the Rhamnolipid

**FIGURE 6:**
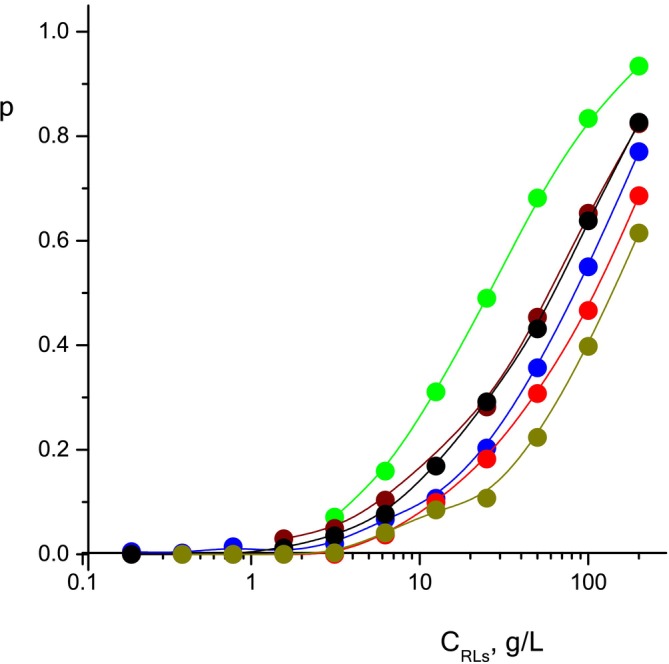
The fraction of solubilized molecules in solutions containing phenol (black), p‐cresol (green), guaiacol (wine), pyrocatechol (blue), resorcinol (red), hydroquinone (dark yellow).

Let us calculate the proportion of solubilized phenol molecules using the ratio (2) experimentally obtained values $D_{S}$ of solubilizers and $D_{\mathrm{RL}}^{\mathrm{mic}}$ diffusion coefficients of micelles of RL, obtained by decomposing the diffusion decay of methylene protons into two exponentials, the data are presented in the table, depending on the concentration of surfactant in the solution:

(3) $$p=\frac{D_{S}^{\text{free}}-D_{S}^{\mathrm{mic}}}{D_{S}^{\text{free}}-D_{S}},$$

assuming: a) $D_{S}^{\text{free}}$ equal to $D_{S}$, measured in the absence of surfactants or with С_surf_<CMC; b) $D_{S}^{\mathrm{mic}}$ equal to $D_{\mathrm{RL}}^{\mathrm{mic}}$. The calculation results are given in Table 1 and, for clarity, are presented in Figure 6. Micellar solubilization processes are predictably absent when С_RL_<CMC; with further increase in the concentration of the RL in all solutions, the proportion of solubilized molecules increases, reaching 0.6–0.9.

**TABLE 1:** Diffusion coefficients of phenol molecules and micelles of RL, fraction of solubilized molecules p, micelle‐water partition coefficient *K*_*m*_, and MSR of phenols depending on the concentration RL in solutions.

С_RL_, g/L	1.56	3.12	6,25	12.5	25	50	100	200
p‐Cresol								
$D_{S}$·10^11^, m^2^/s	79^±3^	76^±3^	69^±3^	58^±2^	44^±2^	29^±1^	17^±1^	7.0^±0.3^
$D_{\mathrm{RL}}^{\mathrm{mic}}$·10^11^, m^2^/s	—	7.8^±0.3^	7.5^±0.3^	7.0^±0.3^	6.3^±0.3^	5.3^±0.2^	4.0^±0.2^	2.0^±0.1^
р, %	—	7^±0^.^4^	16^±1^	31^±2^	49^±3^	68^±4^	83^±5^	93^±5^
*K*_*m*_,%	—	8^±0^.^4^	19^±1^	45^±3^	96^±5^	210^±12^	500^±30^	1420^±80^
MSR·10^2^	—	78^±5^	82^±6^	77^±5^	60^±4^	41^±3^	25^±2^	14^±1^
Guaiacol								
$D_{S}$·10^11^, m^2^/s	78^±3^	76^±3^	72^±3^	68^±3^	60^±2^	47^±2^	31^±1^	16^±1^
$D_{\mathrm{RL}}^{\mathrm{mic}}$·10^11^, m^2^/s	6.6^±0.3^	7.4^±0.3^	7.8^±0.3^	7.6^±0.3^	7.3^±0.3^	6.4^±0.3^	4.8^±0^.^2^	2.3^±0^.^1^
р, %	3^±0^.^2^	5^±0^.^3^	10^±0^.^6^	19^±1^	28^±2^	45^±3^	65^±4^	82^±5^
*K*_*m*_,%	2^±0^.^1^	5^±0^.^3^	12^±0^.^7^	22^±1^	39^±2^	83^±5^	188^±11^	468^±26^
MSR·10^2^	68^±5^	48^±3^	47^±3^	39^±3^	30^±2^	24^±2^	17^±1^	11^±1^
Phenol								
$D_{S}$·10^11^, m^2^/s	91^±4^	87^±3^	83^±3^	76^±3^	66^±3^	53^±2^	35^±1^	17^±1^
$D_{\mathrm{RL}}^{\mathrm{mic}}$·10^11^, m^2^/s	7.2^±0.3^	8.4^±0.3^	8.1^±0.3^	7.6^±0.3^	7.7^±0.3^	6.1^±0.3^	4.4^±0^.^2^	2.1^±0^.^1^
p, %	1.2^±0^.^1^	3.5^±0^.^2^	8^±0^.^4^	17^±1^	29^±2^	43^±3^	64^±4^	83^±5^
*K*_*m*_,%	1.2^±0^.^1^	3.6^±0^.^2^	8.3^±0^.^5^	20^±1^	41^±2^	75^±4^	176^±10^	477^±27^
MSR·10^2^	37^±3^	45^±3^	46^±3^	48^±3^	41^±3^	30^±2^	22^±2^	14^±1^
Pyrocatechol								
$D_{S}$·10^11^, m^2^/s	80^±3^	79^±3^	75^±3^	72^±3^	65^±3^	54^±2^	39^±2^	20^±1^
$D_{\mathrm{RL}}^{\mathrm{mic}}$·10^11^, m^2^/s	6.5^±0.3^	7.5^±0.3^	7.6^±0.3^	7.8^±0.3^	7.2^±0.3^	6.5^±0.3^	5.1^±0^.^2^	2.6^±0^.^1^
р, %	0.4^±0^.^02^	2^±0^.^1^	7^±0^.^4^	11^±0^.^6^	20^±1^	36^±2^	55^±3^	77^±4^
*K*_*m*_,%	0.4^±0^.^02^	2^±0^.^1^	7^±0^.^4^	12^±0^.^7^	26^±1^	56^±3^	122^±7^	335^±19^
MSR·10^2^	10^±1^	22^±2^	34^±2^	26^±2^	24^±2^	21^±2^	16^±1^	11^±1^
Resorcin								
$D_{S}$·10^11^,m^2^/s	78^±3^	78^±3^	75^±3^	71^±3^	65^±3^	56^±2^	44^±2^	26^±1^
$D_{\mathrm{RL}}^{\mathrm{mic}}$·10^11^, m^2^/s	—	—	8.0^±0.3^	7.7^±0.3^	7.3^±0.3^	6.5^±0.3^	5.1^±0^.^2^	2.6^±0^.^1^
р, %	—	—	4^±0^.^2^	10^±0^.^6^	18^±1^	31^±2^	47^±3^	69^±4^
*K*_*m*_, %	—	—	4^±0^.^2^	11^±0^.^6^	22^±1^	44^±2^	87^±5^	219^±12^
MSR·10^2^	—	—	19^±1^	24^±2^	22^±2^	18^±1^	14^±1^	10^±1^
Hydroquinone								
$D_{S}$·10^11^, m^2^/s	81^±3^	80^±3^	78^±3^	74^±3^	73^±3^	64^±3^	50^±2^	33^±1^
$D_{\mathrm{RL}}^{\mathrm{mic}}$·10^11^, m^2^/s	—	7.8^±0.3^	8.0^±0.3^	8.1^±0.3^	7.5^±0.3^	6.8^±0.3^	5.4^±0^.^2^	2.9^±0^.^1^
p, %	—	0.3	4^±0^.^2^	8^±0^.^4^	11^±0^.^6^	22^±1^	40^±2^	62^±4^
*K*_*m*_,%	—	0.3^±0^.^02^	4^±0^.^2^	9^±0^.^5^	12^±0^.^7^	29^±2^	66^±4^	160^±9^
MSR·10^2^	—	3^±0.2^	20^±1^	20^±1^	13^±1^	13^±1^	12^±1^	9^±1^

The efficiency of solubilization by mixed micelles of mono‐RL and di‐RL [63, 64, 65] with each other or with other surfactants [66, 67, 68] also depends on the presence of fatty acid fragments, which, when incorporated into micelles, create competition for solubilizate molecules. By using RL purification from impurities, including residues of fatty acid fragments, it is possible to achieve greater efficiency of the process.

Based on the obtained values of the proportions of solubilized phenol molecules, we calculated the frequently used solubilization characteristics micelle‐water partition coefficient *K*_*m*_ and molar solubilization ratio (MSR), their values are also presented in Table 1.

Мicelle‐water partition coefficient *K*_*m*_ is equal to the ratio of the number of moles of solubilizate in micelles to the number of moles of solubilizate in the aqueous phase:

(4) $$K_{m}=\frac{p}{1-p},$$

MSR, equal to the ratio of the molar concentration of solubilized molecules ${С}_{\mathrm{sol}}^{\mathrm{mic}}$ to the molar concentration of surfactant in the micellar state ${С}_{\text{surf}}^{\mathrm{mic}}$:

(5) $$\mathrm{MSR}=\frac{{С}_{\mathrm{sol}}^{\mathrm{mic}}}{C_{\text{surf}}^{\mathrm{mic}}}=\frac{p\cdot {С}_{\mathrm{sol}}^{\text{total}}}{C_{\text{surf}}^{\text{total}}-\mathrm{CMC}},$$

where ${С}_{\mathrm{sol}}^{\text{total}}$ and ${С}_{\text{surf}}^{\text{total}}$ are total molar concentrations of solubilizate and surfactant in solution. The molar mass of RL was taken to be 650 g/mol, CMC value was taken to be 0.4 g/L.

As can be seen from the table, the solubilization parameters of rhamnolipid are higher for phenols with one hydroxyl group: phenol, p‐cresol, guaiacol, than for phenols with two hydroxyl groups: pyrocatechol, resorcinol and hydroquinone. Obviously, the presence of two hydroxyl groups in phenol molecules, providing hydrogen bonds with water molecules, increases their hydrophilicity and solubility in water and, accordingly, reduces lipophilicity and their solubilization by RL micelles.

Studies of the solubilization of phenol and its compounds carried out by various methods, including NMR, generally reveal the same trends. In solutions of phenol in nonionic surfactants of polyethoxylated nonylphenols [14], the *p* value changes from ~0.25 to ~0.8 with an increase in the surfactant concentration from 0.01 to 0.1 M. The method of micellar‐enhanced ultrafiltration [19] gives a separation efficiency of phenolic compounds from 30 to 70% depending on the concentration and type of the used cationic, anionic, or nonionic surfactant. Aggregates of cetyltrimethylammonium bromide (CTAB) and an oppositely charged copolymer of maleic acid and styrene have a high capacity and give high values of distribution constants of phenols between the aqueous phase and aggregates [20]. Sabatino et al. [69] are obtained for the solubilization of phenol with CTAB micelles at normal acidity p ≈ 40%; with increasing pH, the coefficient p increases to 72% at pH = 12.31. Solubilization of phenol molecules, both in their undissociated and dissociated states, with cationic dioctadecyl dimethylammonium chloride vesicles were investigated using NMR techniques [57], the value of the phenol bound fraction obtained was on average about 40% with a change in phenol concentration from 3 to 10 mM. Taking into account the results of the authors' previous works [48, 49, 50], it can be concluded that the extraction properties of rhamnolipid biosurfactants are not inferior to the properties of synthetic surfactants. At the same time, biosurfactants have undoubted advantages, possessing low toxicity, high biodegradability, the ability to degrade organic compounds, and can be considered as an alternative to synthetic surfactants in the development of technologies for the treatment of industrial wastewater using micellar solubilization of organic pollutants.

The wide range of rhamnolipid concentrations (0.05–200 g/L) made it possible to answer the question of how its solubilization properties change with increasing concentration during the transition from the monomeric state C_RL_<CMC to spherical, cylindrical micelles, vesicles at C_RL_>CMC [38]. Typical values of rhamnolipid concentrations used for water purification from heavy metals and organic impurities using the MEUF procedure are within the range of 0.5–1.75 g/L [43, 47, 70], and C_RL_ = 0.025% is used for soil remediation [40, 71]. Our concentration studies will be useful for researchers and practitioners working with biosurfactants, micellar solubilization, and green remediation technologies.

## 4 Conclusions

The NMR diffusometry method with a pulsed magnetic field gradient and Fourier transform of the spin echo signal allows selective measurement of the diffusion coefficients of molecules in multicomponent systems, such as surfactant solutions. As applied to the problem of micellar solubilization, the NMR diffusometry method makes it possible, by comparing the diffusion coefficients of surfactant micelles and solubilizate molecules, to determine the solubilization properties of surfactants. In particular in this work, we compared the biological surfactant rhamnolipid with respect to phenolic compounds with one and two hydroxyl groups: phenol, p‐cresol, guaiacol and pyrocatechol, resorcinol, hydroquinone, respectively.

Within the framework of the model of two states of solubilizate molecules in solution—free in the aqueous phase and bound in the composition of surfactant aggregates—the solubilization characteristics of rhamnolipid were calculated, specifically, the proportion of solubilized molecules p, micelle‐water partition coefficient *K*_*m*_, and MSR depending on the concentration of rhamnolipid in solution. The solubilization properties of rhamnolipid remain quite high for both phenols with one hydroxyl group and those with two hydroxyl groups. The proportion of solubilized phenol molecules in all solutions increases with increasing of the RL concentration, reaching *p* = 0.6–0.9 at C_RL_ = 200 g/L. The solubilization properties of rhamnolipid micelles are higher for phenols with one hydroxyl group than for more hydrophilic phenols with two hydroxyl groups. Taking into account the low toxicity and high biodegradability of biosurfactants, solubilizing organic molecules by rhamnolipid micelles (phenols and phenolic compounds, substances of the BTEX group and naphthalene) in combination with the ultrafine filtration technique [35, 72] is an approach that can be used to develop “green,” low‐toxic, low‐cost methods of water purification, including purification of industrial wastewater.

### Peer Review

The peer review history for this article is available at https://www.webofscience.com/api/gateway/wos/peer‐review/10.1002/mrc.5530.

## Supporting information


**FIGURE S1** Diffusion decays of p‐cresol at different RL concentrations (g/L) in D_2_O solutions.
FIGURE S2 Diffusion decays of pyrocatechol at different RL concentrations (g/L) in D_2_O solutions.
FIGURE S3 Diffusion decays of resorcinol at different RL concentrations (g/L) in D_2_O solutions.
FIGURE S4 Diffusion decays of phenol at different RL concentrations (g/L) in D_2_O solutions.

## Data Availability

The data that support the findings of this study are available from the corresponding author upon reasonable request.
