# Supplementary material for: Micellar Solubilization of Phenols With One or Two Hydroxyl Groups Using Biological Surfactant Rhamnolipid
Source: Magn Reson Chem. 2025 May 19;63(7):508–17. doi: 10.1002/mrc.5530 (PMC12129644; doi:10.1002/mrc.5530)
Supplement: Supplementary file 1 — FIGURE S1 Diffusion decays of p‐cresol at different RL concentrations (g/L) in D2O solutions. FIGURE S2 Diffusion decays of pyrocatechol at different RL concentrations (g/L) in D2O solutions. FIGURE S3 Diffusion decays of resorcinol at different RL concentrations (g/L) in D2O solutions. FIGURE S4 Diffusion decays of phenol at different RL concentrations (g/L) in D2O solutions. [file MRC-63-508-s001.docx]

**Supporting materials**

**Micellar solubilization of phenols with one or two hydroxyl groups using biological surfactant rhamnolipid**

**Victor P. Arkhipov,^1^ Ruslan V. Arkhipov,^2^ Andrei Filippov^3^***

^1^Department of Physics, Kazan National Research Technological University, 420015, Kazan, Russian Federation

^2^Institute of Physics, Kazan Federal University, 420008 Kazan, Russian Federation

^3^Chemistry of Interfaces, Luleå University of Technology, SE-97187 Luleå, Sweden

Correspondence

Andrei Filippov, Chemistry of Interfaces, Luleå University of Technology, Luleå, Sweden.

Email: [andrei.filippov@ltu.se](mailto:andrei.filippov@ltu.se)

Figure S1 Diffusion decays of p-cresol at different RL concentrations (g/L) in D_2_O solutions.

Figure S2 Diffusion decays of pyrocatechol at different RL concentrations (g/L) in D_2_O solutions.

Figure S3 Diffusion decays of resorcinol at different RL concentrations (g/L) in D_2_O solutions.

Figure S4 Diffusion decays of phenol at different RL concentrations (g/L) in D_2_O solutions.
